# A Treatise on the Diseases of the Heart and Great Vessels, and on the Affections Which May Be Mistaken for Them; Comprising the Author's View of the Physiology of the Heart's Action and Sounds

**Published:** 1839-10

**Authors:** 


### Art. X.

*A Treatise on the Diseases of the Heart and Great Vessels, and on the Affections which may be mistaken for them; comprising the Author's View of the Physiology of the Heart's Action and Sounds, as demonstrated by his experiments on the motions and sounds in* 1830 *and on the sounds in* 1834-5. By J. Hope, m.d. f.r.s. *Third Edition, corrected and greatly enlarged.—London.* 8vo, pp. 639.

Since the period of the first publication of Dr. Hope's Treatise in 1832, it has been so widely disseminated, through the medium of two London editions and several foreign editions and translations, and by the transference of its most valuable matter to the pages of the Cyclopædia of Practical Medicine, that it is quite unnecessary, on the present occasion, to occupy our pages with any account of the general plan and character of the work. We shall therefore confine our notice to the new portions of the present edition. It is right to state, however, that this is by no means to be regarded as a mere republication of the previous treatise. From the careful revision which its whole contents have undergone, and from the addition of at least a third of new and valuable matter, the present may, in reality, be considered rather a new work than a new edition.

The following is the author's enumeration of the additions; and we shall find it convenient to examine them in the same order:

"1. The natural sounds of the heart. 2. The sound of costal percussion, with or without tinnitus. (*Laennec's Cliquetis.*) 3. Murmurs from valvular disease, and the whole subject of particular valvular diagnosis, which will now, I confidently hope, be found one of the most simple and easy departments of auscultation. 4. Murmurs of the heart and arteries independent of organic disease. 5. Venous murmurs. 6. Musical murmurs. 7. Abdominal murmurs, both connected with pregnancy and otherwise. 8. Tremor or thrill of the heart, arteries, and veins. 9. Signs, general and physical, of pericarditis and endopericarditis. 10. Connexion of diseases of the heart with apoplexy, palsy, &c. 11. Partial dilatation or real aneurism of the heart. 12. The signs, physical and general, and the pulses of softening. 13. Signs of adipose disease of the heart. 14. Aneurisms of the aorta bursting into the pulmonary artery and right ventricle. 15. Abdominal aneurisms. 16. Anæmic, nervous, dyspeptic, plethoric, bilious, and other sympathetic affections of the heart, with their diagnosis. 17. Displacements. 18. The pulses of disease of the heart." (Preface, pp. v.-vi.)

The Natural Sounds of the Heart. The additional matter introduced into the present volume, under this head, consists of an elaborate examination of the immediate causes of the two sounds. No alteration we think is made in that part relating to the motions of the organ with which the sounds correspond; this part of the subject being so well illustrated and confirmed by experimental evidence, as to preclude all contradiction or doubt. We think also that there can hardly be any difference of opinion as to the cause of the *second* sound; the result of all the numerous experiments going to prove that this sound is caused by the reaction of the blood on the semilunar valves during the ventricular diastole, as first announced by Dr. Carswell.

Dr. Hope attributes the first sound to three causes: 1, "*the sound of muscular extension;*" 2, "*bruit musculaire ou rotatoire,*—the dull, rumbling sound of muscular contraction;" 3, "*the sound of valvular extension*," the most important, he says, of the three. We will briefly recapitulate the evidence on which Dr. H. grounds these opinions.

1. By the term "muscular extension," he says that "he means a loud, smart sound, produced by the abstract act of sudden, jerking extension of the already braced muscular walls, at the moment when the auricular valves close; in the same way that when the valve of a pair of bellows closes, its leather is put on the stretch, and if not rigid produces sound." This sound differs from *bruit musculaire*, inasmuch as it may be produced in dead muscle, in which bruit musculaire obviously could not exist. The grounds on which this sound of extension has been adopted are the following: *a*. In Dr. Hope's experiments, and in those of the Dublin and London Committees of the British Association, the walls of the ventricles, during each systole, became suddenly tense and hard to the touch, and the tension and hardness exactly coincided with the first sound. *b*. The impulse from lateral expansion was greatest at the margin of the auricular orifices, there throwing the finger out with a violent jerk. *c*. When the resistance of the auricular valves was removed, and the sudden jerk of muscular extension prevented, the first sound was dull and obscure, like the common muscular sound.

2. The existence of bruit musculaire, as one of the causes of the first sound, is proved by the experiment in which the auricular valves were destroyed, the sound then becoming dull and obscure.

3. Under the term valves, Dr. Hope includes the chordæ tendineæ. The grounds for assuming that valvular tension is one of the causes of the first sound, are: *a*. The evidence offered above in favour of muscular extension. *b*. The London Committee state that they *felt* the chordæ tendineæ of the mitral valve become *tense in systole*, and lax in diastole. (Exp. 12.) *c*. That sound can be produced by valvular reaction is proved by the second sound, which is universally admitted to be generated by smaller membranes, acted upon with inferior force. *d*. There are certain pathological conditions of the heart, as dilatation with attenuation, in which the first sound becomes as pure a *click* as the second, and is, therefore, probably due to the extension of the auricular valves and chordæ tendineæ alone, the feeble contractions being insufficient to produce bruit musculaire or muscular extension.

The first sound, then, according to Dr. Hope's view, is a compound sound, depending on several causes, the respective and relative influence exercised by which, in producing the modifications in its character, he thus describes:

"The valvular click gives smartness and intensity to the commencement of the first sound, and in feeble hearts, in which the sounds of extension and of bruit musculaire are absent, the click alone is heard, causing the first sound to be identical in quality with the second. This occurs, for instance, in dilatation with attenuation. The sound of muscular extension superadds bluntness and loudness to the valvular click, and is probably a principal cause of the extraordinary intensity of the first sound, often observed in violent palpitation. The *bruit musculaire* forms a gradually diminishing prolongation of the sound to the end of the act of contraction; but when the heart acts feebly, either from disease or from mere temporary exhaustion or faintness, the *bruit musculaire* may be partially or wholly absent." (p. 63.)

This is all very ingenious; but it does not carry complete conviction to our minds any more than the explanation of Dr. Williams, which we shall here also introduce, in order that our readers may see at once the theories of the two highest authorities on this subject. We would at the same time remark that the two hypotheses seem to us to differ rather in terms than in point of fact; or to differ only in the relative degree of importance attached by the two writers to the different causes. We confess we cannot accurately discriminate Dr. Hope's "muscular extension," as above explained by himself, from Dr. Williams's "sudden tightening of the muscular fibres on the mass of blood." With whom the credit of the explanation rests is another question, on which we will not touch. Dr. Williams says:

"By excluding the blood we are thus brought to the conclusion that the cause of the sound must be in the solid structure of the ventricles: it is our next question whether it be in any part of them in particular. Several writers have ascribed it to the auriculo-ventricular valves, which, when they close, are supposed to produce a flapping sound. But the act of closing these valves is momentary, and takes place only at the commencement of the systole; whereas the first sound of the heart is prolonged through its whole duration. Further, in some of our experiments the first sound continued, although impaired, when the auriculo-ventricular valves were prevented from acting, by fingers introduced into their orifices, or by some of their cords being cut. Still these valves may produce a part of the sound, for at each contraction they are suddenly tightened, in a manner calculated to generate sound. But are the valves the only parts which are tightened at each systole of the ventricles? Is not every muscular fibre in the ventricles suddenly tightened by this action? Here are the elements of sound, motion vigorous and rapid, suddenly resisted by the mass of blood to be urged forwards by the contraction, and the contracting motion and the resistance, although greatest at first, continue to act as vibrating forces during the whole systole; hence the prolongation of the sound. . . . . In my experiments there was the best proof that we could have, that the muscular contraction of the heart produced systolic sound, for we had the heart out of the body, without its blood, without valvular action, lying on the table, or on my hand, and its contractions were still accompanied with a sound, weak, indeed, but in character resembling its natural first sound. The walls of the ventricles appear to be peculiarly calculated to generate sound; their flaccid state when relaxed, the fineness of their fibres, and the harmony with which they suddenly contract on their contents, and become almost as hard as a stone (as we can feel in the living heart of a stunned animal), fulfil the conditions best calculated for the production of sound. The commencement of the systole, producing the tightening of the auricular valves, and thereby completing the resistance of the body of blood on which the contracting fibres have to act, is naturally its loudest part, and often has a flapping character; that which continues after is more dull, and is prolonged according to the quantity of blood to be expelled, and the continued strength of the contraction."*

The London Committee, after Magendie, have admitted the impulse of the apex against the thoracic parietes, as an occasional source of augmentation to the first sound. Dr. Hope denies that this can have any part in the production or modification of the sound. The following are the considerations by which he thinks this opinion is discountenanced: *a.* We are not in possession of any experimental evidence that the apex ever actually impinges against or strikes the chest. *b.* Since the organ is maintained in apposition with the walls of the chest, by a force equivalent to fifteen pounds to every square inch of its surface, by atmospheric pressure (as first remarked by Mr. Bryan), it is reasonable to infer, in the absence of proof to the contrary, that it never quits its contact with the walls, and, consequently, can never *strike against* them. *c.* The first sound is rendered louder by leaning forwards, and to the left side, and by

* Lectures on Diseases of the Chest, delivered in 1836-7; reprinted from the *Med. Gazette,* Lect. xxiv. pp. 154-5.

a full expiration, which have the effect of determining the heart, if possible, to still more complete *apposition with* the walls of the chest, a condition incompatible with increased *impulse against* it. Although we think there is much force in these objections, we cannot admit that they *entirely* disprove the proposition, as maintained by the London Committee.

Metallic Tinkling. Dr. Hope states that the motion of the heart creates an occasional sound by striking against the inferior margin of the fifth rib, which is attended with *cliquetis*, or metallic tinkling, when the impulse is smart. This, he says, may be prevented at pleasure by pressing the edge of the stethoscope, or anything else, into the intercostal space, by which that space is put, internally, on the same plane as the rib, over which the heart then glides without catching. This phenomenon, which Dr. Hope supposes to be identical with the metallic tinkling of Laennec, is only met with in meager persons (as remarked by Laennec and Bouillaud), because in the well-conditioned the intercostal spaces are full and resistent, and, consequently, the edge of the rib is not exposed. The following extract contains the result of Dr. Hope's observations on this subject.

"Carrington consulted me, March 30, 1838, æt, 30, tall, thin, a footman; has hypertrophy, with palpitation, and dyspnœa on exertion. There is pretty strong impulse between the fifth and sixth left ribs, where the apex impinges. On placing the stethoscope immediately over this spot, a metallic tinnitus was heard, exactly like that produced by tapping the back of the hand with the finger, while the palm covers the ear. The first sound of the heart seems to be *double*, like that produced by tapping a table with two fingers at once; the second of the two sounds was the tinnitus. I have for many years noticed this double sound with tinnitus. I made the following series of observations on the phenomenon: 1. The tinnitus ceased and the sound was single when either the upper or lower edge of the stethoscope was pressed obliquely into the intercostal space. 2. The tinnitus ceased but the sound continued double when the stethoscope, with the stopper in, was applied flatly over the ribs. 3. I filled the hollow cone with cotton wadding, which, by its elasticity, pressed the intercostal space inwards, when the tinnitus ceased and the sound was single. 4. When I withdrew half the wadding and left the cone only lightly filled, the double sound and tinnitus returned, though rather diminished. 5. The tinnitus continued, though rather duller, when I placed a penny flat across the two ribs, and listened with the stethoscope upon it. 6. It ceased, as well as the double sound, on full inspiration; and was always strongest during expiration. 7. It was increased by leaning forwards during expiration." (p. 602.)

Dr. Hope devotes a short space to a refutation of the *alternating* theory of the heart's movements, originally proposed by M. Magendie, and adopted by M. Bouillaud, Dr. Bostock, and others. This view, which supposes that the contraction of the auricles alternates with that of the ventricles, and *vice versâ*, is obnoxious to the physiological objection of leaving no interval of perfect repose for the whole organ, and is refuted by Dr. Hope's first series of experiments. The source of fallacy consists in M. Magendie's having operated on *living* animals, in which the action of the heart is so "violent, convulsive, and rapid," as to encroach materially on the interval of repose, and consequently to prevent the deduction of accurate conclusions. Dr. Hope justly remarks that no safe inferences can be drawn from the movements observed, unless the animal be completely deprived of sensibility at the time. (p. 61.)

We are sorry to learn from the work of Dr. Hope that some difference has arisen between him and Dr. Williams, with regard to the experiments on animals on which their respective doctrines of the sounds of the heart are founded; Dr. Hope preferring an exclusive claim to the institution and first performance of these experiments, the justice of which Dr. Williams does not admit. We purposely avoid entering into the merits of this dispute, but we cannot help expressing our regret that Dr. Hope has not used more dignified language in the assertion of what he deems his rights. We cannot but think, also, that Dr. Hope betrays, on several occasions in this volume, greater sensitiveness for his reputation as a discoverer than becomes one whose great merits are so readily acknowledged by his professional brethren in every country where auscultation is known and practised.

Valvular Diagnosis. On this subject Dr. Hope has supplied us with more original and useful matter than, perhaps, on any other. If the grounds on which he has established his rules be sound, valvular diagnosis may be considered to be reduced to a degree of facility and precision unequalled by that of any other disease of the heart. We cannot withhold our expressions of admiration at the great attention which Dr. Hope has given to this subject, and the indefatigable perseverance with which he has worked out a problem so difficult and obscure. As these rules have not previously been published in a complete and authentic form, and are probably new to the great majority of our readers, we shall not deem a somewhat lengthened analysis of them an unprofitable occupation of our space.

Three questions have been raised on this subject, to which it is advisable briefly to advert in the first place: these have regard, 1, to the *possibility*, 2, the *facility* or *general practicability*, and, 3, the *utility* of differential diagnosis of valvular disease. We shall notice them in order.

1. Both Laennec (*Forbes's 4th Edit.* p. 519,) and Andral (*Spillan's Trans. of Clin. Med.* p. 280,) state that they have met with murmurs without diseased orifices, and with diseased orifices which produced no murmur; and M. Piorry says (*Bouillaud*, vol. i. p. 174,) that he has not detected a murmur in one twentieth of his cases of valvular disease. Drs. Graves and Stokes (*Dublin Journal*, vol. xiv. p. 180,) repeat the assertions of Laennec and Andral, term valvular diagnosis "the most difficult part of medicine," and state that physical signs, taken alone, are in no case sufficient for its accomplishment; and even promise hereafter to "bring abundant proofs" that "*all varieties* of valvular murmur may occur without organic disease." On the other hand, Dr. Hope states that the diagnosis may be made with "almost complete certainty," (p. 382;) M. Bouillaud has never met with a single instance in more than 100 cases of valvular disease, in which a murmur has not been detected by careful examination, (*Traité*, vol. i. p. 175); Dr. Corrigan has never met with an instance of "narrowing of the cardiac orifices" unattended by a murmur, (*Dublin Journal*, vol. x. p. 174); and Dr. Elliotson had, at the time of delivering the Lumleyan lectures, always detected a murmur, except in one instance of mitral contraction, (*Lum. Lect.*, p. 19.) Now, since negative evidence will not bear comparison, as to value, with positive evidence, and since the positive evidence is here so abundant and decisive, it is clear that there must have been some lurking sources of fallacy in the former class of these discrepant statements.

The errors of diagnosis have probably arisen from some of the following causes: *a.* It is very possible that a murmur may exist, but escape detection from imperfect exploration, ignorance of the exact situation of the valves, extraneous sounds produced by the process of immediate auscultation, &c. *b.* It is admitted by Dr. Hope and proved by his cases that contraction of the mitral orifice frequently exists without murmur. *c.* Mere dilatation of a cavity or contraction of the chordæ tendineæ, without disease of the orifice in particular, may cause regurgitation; cases of which are given by Dr. Hope. *d.* Even examination after death cannot always be depended upon unless it is conducted with especial care. Thus, in one of the cases of which Dr. Hope has given a plate, he foretold disease of the aortic valves, and the patient subsequently died under the care of a practitioner out of town. "The gentleman who made the examination informed me," says Dr. H., "that I had committed an error in diagnosis, the aortic valves being sound and the mitral diseased. The preparation was sent to me, and he had mistaken the aortic for the mitral valve." (p. 621.) *e.* The diagnosis of valvular murmurs from attrition, and other murmurs, has hitherto been imperfectly understood.—These are some of the circumstances which may account for the discrepancies in the statements of different practitioners in valvular diagnosis. The rules now offered by Dr. Hope will, we think, go far to prevent the occurrence of any such discordances among future observers.

2. With regard to the *facility* with which the diagnosis of valvular disease may be acquired, we give it as our decided opinion that there is scarcely any other disease in which accuracy is so attainable. We have seen so many illustrations of the truth of this assertion, that we wish the greatest confidence to be placed in it. As a demonstration of the accuracy with which even the most complex case may be unravelled, we refer our readers to the important case of Goff in this work, (pp. 610, 625,) in which the diagnosis of a young student, in a singularly complicated assemblage of signs and morbid alterations, was submitted to the infallible test of dissection, and perfectly confirmed in every particular.

3. As to the *value* of minute valvular diagnosis, we think no scientific physician can entertain a doubt, any more than he can entertain a doubt of the value of accuracy in the diagnosis of diseases in general.

Valvular murmurs are distinguished from other murmurs, and murmurs seated in different valves are distinguished from each other by the motion of the heart which they accompany, by their situation, their character, the pulse with which they coincide, by certain peculiarities in the disease from which they are to be distinguished, and by the general symptoms with which they are attended.

Each valve or orifice of the heart may be the seat of two distinct murmurs: one accompanying the systole of the ventricles, the *systolic murmur;* the other accompanying the diastole, the *diastolic murmur.* The systolic murmur in the semilunar valves is *direct;* that is, it arises from a contraction of the orifice, or an impediment to the onward progress of the blood through the orifice. The systolic murmur in the auriculo-ventricular orifices is *regurgitant,* depending either on enlargement of the orifice, so that the valve, of normal proportions, cannot completely close it, or upon corrugation, nodulation, ossification, or some other form of disease of the valve itself, preventing it from attaining perfect apposition, and producing permanent patency of the orifice, so that partial regurgitation of blood is permitted during the ventricular contraction. The *diastolic* murmur is, of course, the reverse of the systolic, being *regurgitant* in the sigmoid valves, and *direct* in the auriculo-ventricular; which latter is the most rare of all murmurs. Any of these murmurs may result from the various diseases to which the valves are subject. The following is Dr. Hope's account of the situations in which valvular murmurs are most audible, by listening in which situations, he says, they may be distinguished from each other, and referred to their respective causes with facility and accuracy.

"Murmurs situated in the semilunar valves are best heard immediately over those valves, (that is, on the sternum, opposite to the inferior margin of the third ribs, when the patient is horizontal, and a little lower when he is erect,) and thence for about two inches upwards, along the diverging courses of the aorta and pulmonary artery respectively. [The former diverging to the right second intercostal space, the latter to the left.] A distinct murmur, high up the aorta, proceeds from the aortic valves, as a pulmonic murmur is only feebly and indistinctly transmitted in that direction. It may be known that the murmur proceeds from the aortic valves, rather than from the diseased ascending aorta itself, by its key not being higher than a whispered *r*, whereas a murmur from the aorta itself is commonly a tone or two higher, approaching towards an *s*, and also seems much nearer and more superficial.

"A distinct murmur, high up the pulmonary artery, proceeds from the pulmonic valves, as an aortic murmur is only feebly and indistinctly transmitted in that direction. The pulmonic murmur, whether situated in the valves or the artery itself (as when dilated), always sounds near and superficial, provided the current be sufficiently strong, because the valves and the artery are close to the surface, the valves being not only in front of the aortic valves, but half an inch higher up. A murmur in the pulmonic orifice is more audible down the tract of the right ventricle than the left, which is a corroborative circumstance.

"Thus, by listening *high up* the aorta [second intercostal space, right of the sternum,] and pulmonary artery, [second intercostal space, left of the sternum,] it is easily ascertained in which vessel the murmur is seated. This rule will even apply to semilunar regurgitations, notwithstanding that these murmurs are weaker and not so well transmitted *up* the vessels in consequence of the current setting *out of* them into the ventricles. There is a further and most important advantage in exploring murmurs of the semilunar valves high up the vessels; namely, that in these situations murmurs of the auricular valves are, from their remoteness, either wholly inaudible or very obscure; although, therefore, an auricular murmur should coexist, it would not prevent the auscultator from deciding that a loud and near-sounding murmur, heard high up the vessels, was generated in or above the arterial orifices.

"Murmurs seated in the auricular valves are best heard at that part of the precordial region where, from the heart being in contact with the walls of the chest, there is dulness on percussion—in short, about the apex; for the murmur is best conducted to the surface, through a solid medium. The upper and *left* side of the dull portion being nearest to the mitral valve, is the best point for exploring its murmurs; and this point will generally be found situated about the fifth rib or subjacent intercostal space, and a little to the right of the nipple: in females it is under the mamma, when pretty well raised, and a little to the right of its centre. If the impulse of the heart be perceptible, there is no better guide than this to the situation in question. The auscultator has only to place his stethoscope about an inch above the spot where the apex impinges.

"The upper and *right* side of the dull portion being nearest to the tricuspid valve is the best point for exploring the murmurs of this valve; and the point will generally be found on or near the sternum, at the same level as on the opposite side. If, in making these explorations of either valve, the stethoscope be placed half over the dull portion and half over the thin resonant edge of the lung, the object will be sufficiently answered.

"There is a further and most important advantage in exploring murmurs of the auricular valves in these low situations: namely, that the murmurs sound so *near* and distinct as to preclude the idea of their being generated in the arterial orifices, the murmurs of which always sound *remote* and obscure when explored near the apex of the heart.

"When both the semilunar and auricular valves are diseased it is perfectly easy to ascertain this by observing, according to the above rules, together with those for the pitch of murmurs, that there are two distinct sources of sound.

"When two murmurs are situated in the same orifice, this is readily ascertained by tracing them up to the single source, and noticing that one attends the first and the other the second sound." (p. 90.)

In order perfectly to comprehend these rules it is requisite that the auscultator possess an accurate knowledge of the relative anatomy of the parts. The plate facing the title-page and the diagrams (fig. 4, A, B, and C, p. 615,) are admirably adapted to show the sources and relations of these murmurs, and will tend not a little, we think, to simplify this apparently complex and difficult subject.

The *pitch* or *key* of the murmurs in the several valves varies so greatly that, in a great majority of cases, it constitutes a valuable aid in diagnosis. The following is the author's description of these varieties:

"The bellows, sawing, rasping, and continuous murmurs in the heart are louder, *cæteris paribus*, in proportion as the stream of blood through the contracted orifice is stronger. This, which is obvious, on theoretical grounds, I have found amply confirmed by observation. Thus, murmurs are increased by accelerating the heart's action, and diminished by calming it, especially if the pulse be much lowered by digitalis. Again, I have collected six or seven cases of valvular disease, in which there was one strong contraction of the ventricles, producing a pulse, followed by two or three feeble contractions, attended by a barely perceptible pulse. The strong contractions occasioned a murmur—the weak, none. Again, the currents by regurgitation through the sigmoid valves, and those flowing out of the auricles into the ventricles, through the contracted auricular valves, are more feeble than the currents setting in the opposite direction, that is, out of the ventricles; and the corresponding murmurs I have invariably found to be weaker. The strength of the current, however, is not the only circumstance which occasions loudness of murmurs; for such a configuration of the stricture as most breaks the stream produces not only a rougher murmur, as already shown, but one of greater intensity. Accordingly, we find rough murmurs, cæteris paribus, louder than others, with the exception of musical tones, these being, from their acute nature, more calculated for transmission to a distance.

"The pitch or key of the bellows, filing, sawing, or rasping murmur, (as distinguished from their roughness), depend mainly on the depth or distance from the surface at which murmurs are generated, the pitch being higher in proportion as they are nearer, and *vice versâ*; but it is also slightly elevated by a stronger current and depressed by a weaker. A very narrow aperture also raises the key, provided the current be strong. These circumstances were not pointed out by Laennec or the other French writers; whence has resulted the prevailing confusion respecting the meaning of the above epithets, filing, sawing, &c. After much attention devoted to this point, I think that the following characters will be found at once tolerably accurate and easy of comprehension.

"Murmurs seated in the pulmonary orifice or artery, from being the most superficial, are on a higher key than any others. Though they are seldom as high as the whispered letter *s*, yet they range between this and the whispered letter *r*. Murmurs originating in the ascending aorta, where it approaches near to the sternum, are, for the same reason, on almost as high a key.

"Murmurs in the aortic orifice, being rather more deeply seated, seldom rise higher than a whispered *r*, which is their average key, and it is perhaps the most ordinary type of the sawing sound. M. Bouillaud, however, (to whom I am indebted for the ingenious idea of representing sounds by letters, and who has used *s* and *r* for this purpose,) thinks that *s* more truly represents the sawing sound.

"Murmurs from aortic and pulmonic regurgitation, in consequence of the currents being weaker, are generally two tones lower, like whispering *awe* by inspiration; and the click of the valves, when audible, may be represented by prefixing the letter *p*, as *paw*.

"Murmurs in the mitral valve, from being still more deeply seated, are, on the average, four tones lower, like a whispered *who;* the tone is somewhat elevated by a very strong current, as that of violent mitral regurgitation, and depressed by a feeble current, as that producing diastolic mitral murmurs.

"Tricuspid murmurs are rather higher toned than mitral, because less deeply seated." (pp. 84-6.)

Dr. Hope thinks that *musical murmurs* indicate nothing more than common murmurs. Of *continuous murmurs* Dr. Hope says:

"They will probably be found to indicate sometimes organic disease, attended with regurgitation out of the aorta into the right ventricle or pulmonary artery: sometimes churning of a little serum between layers of rough lymph on the pericardium; and sometimes probably dilatation of the pulmonary artery and compression of the vena innominata." (p. 89.)

The *pulse* is a further assistance in the detection of valvular disease, when its characters are fully understood; although, without a thorough comprehension of its varieties and their sources, it must be rather a means of confusing than of aiding the practitioner. The "jerking" pulse of aortic regurgitation, we are told by Dr. Hope, is in particular so characteristic that from its presence alone a correct diagnosis may often be formed of the disease. The original genius of Corvisart endeavoured to connect peculiarities of the pulse with particular diseases of the heart, but he was by no means successful in the attempt. Laennec does not even mention the pulse in his diagnosis of valvular disease; he devotes a considerable portion of one of his earlier chapters to the exposition of his reasons for agreeing with Celsus that the pulse is "res fallacissima." Andral says that the pulse presents so many varieties that "only a secondary importance can be attached to it." M. Bouillaud says, that in valvular disease of the left side the examination of the pulse is a source of assistance which should not be neglected in differential diagnosis; and he straightway proceeds to describe as peculiar to aortic obstruction a pulse which, Dr. Hope assures us, that lesion very rarely occasions, and which is characteristic of mitral disease. Dr. Elliotson gives a much more correct description of the pulse in his Lumleyan lectures, a work which has by no means been estimated at its real value.

We think that Dr. Hope has deprived this subject of much of its obscurity and confusion, and has gone far to prove, by pathological facts, what Bichat inferred from physiological reasoning, that "to every species of action of the heart there corresponds a particular kind of pulse." The following account of the varieties of the pulse in valvular disease we have abstracted from Dr. Hope's work; we think a knowledge of them will prove a very valuable addition to our usual means of diagnosis.

The varieties of the pulse in valvular disease depend on the particular valve in which the disease is seated, and on the nature of the disease itself. 1. *Mitral valve.* Both contraction of this orifice, and regurgitation through it, confer on the pulse various degrees of smallness, weakness, irregularity, inequality, and intermission, proportionate to the amount of disease in a given case. 2. *Aortic valve.—a. Contraction.*

This form of disease must be very great to render the pulse small, weak, irregular, unequal, or intermittent. Dr. Hope has never found it to possess these characters in any remarkable degree unless the valves were either soldered together by cartilaginous degeneration, or more or less fixed by ossification in the closed position, so that the aperture was only a limited chink. An induration of the size of an ordinary pea has little effect on the fullness, firmness, and regularity of the pulse, and slighter degrees of contraction appear to have no effect on it whatever. These facts are curious and singular, but are proved by many of Dr. Hope's cases.—*b. Regurgitation.* Under this head Dr. Hope also includes regurgitation out of the aorta into the right ventricle, or into the pulmonary artery. The pulse is preeminently *jerking*, a high degree of the pulse of unfilled arteries, as seen in anæmia from any cause. The diastole or beat of the artery is short and quick, as if the blood were smartly jerked or shot under the finger, the vessel, during the intervals, feeling unusually empty. It differs from the jerking pulse of anæmia, in being more marked and in not necessarily being frequent, as the anæmic pulse is when its jerk is distinct. 3. *Valvular disease on the right side.* This form of disease produces little effect on the pulse, not being immediately connected with the arterial system, and having little influence over the general action of the organ. (pp. 375-80.)

It only remains to quote the diagnosis of valvular murmurs from those produced by anæmia, pericarditis, and aortic disease; and we shall have completed our analysis of Dr. Hope's present views on this extensive subject. The diagnosis of organic valvular murmurs from anæmic murmurs depends on the following characters of the latter. *a.* They are confined to the aortic orifice. *b.* They occur only with the systolic motion of the ventricles. *c.* They are always weak, and of the soft or bellows kind. *d.* They are generally attended with venous and arterial murmurs and thrill. *e.* They exist only when the heart's action is excited. *f.* They are accompanied with the *jerking* pulse. *g.* They are attended with general anæmic and hysterical or nervous symptoms. *h.* They disappear when the anæmia is cured by iron and animal food. These circumstances should be contrasted with the following characters of organic valvular murmur. *a.* Only the murmur of aortic contraction can be confounded with that from anæmia, by any one who has studied the rules for differential valvular diagnosis. *b.* This disease does nor produce the jerking pulse, venous or arterial murmurs. *c.* Valvular murmurs are often rough. *d.* They persist without intermission, even though the circulation be slow. *e.* They are not necessarily attended with general anæmic or nervous symptoms.

We formerly expressed our concurrence with the statement of M. Bouillaud, that valvular murmurs cannot always be distinguished with certainty from the attrition murmurs of pericarditis, especially when the two classes of sounds coexist in the complicated disease of endo-pericarditis. We are happy to see that Dr. Hope does not share this difficulty with us, and that he has supplied a diagnosis on which he can generally rely with confidence. The management of the complicated cases above mentioned renders the diagnosis sometimes exceedingly desirable, especially with reference to prognosis and the treatment by mercury.

"Some writers," says Dr. Hope, "especially M. Bouillaud, (tom. ii. p. 211,) have experienced great difficulty in descriminating these two classes of sounds. I cannot say that, since I became acquainted with attrition murmurs, I have participated in this difficulty; even when the two classes of sounds existed simultaneously, and each was double. This is mainly from attending to the rules which I have so often inculcated; namely, of listening to murmurs of the sigmoid valves, two inches or more up the aorta or pulmonary artery, where attrition murmurs are almost inaudible, and of listening to murmurs of the auricular valves a little above the apex of the heart, where they are sure to be the loudest, whereas attrition murmurs may be louder at other parts of the heart, where they happen to be generated. Further, attrition murmurs present the following distinctive peculiarities. 1. They are usually of a much rougher quality of sound than the valvular, so that when the two coexist the one may be heard *through* the other. 2. When a murmur with the second sound is rough, as rasping, creaking, croaking, &c., it is certainly from attrition; as I have never known a valvular murmur with the second sound to be rough, the diastolic currents being too feeble to produce roughness. 3. Attrition murmurs are almost always attended with vibratory tremor; whereas valvular murmurs rarely present this phenomenon, and generally in a slighter degree. 4. Attrition murmurs are apt to undergo frequent and sudden changes of character and situation (Stokes), which are very pathognomonic, because valvular murmurs change little in character, and not at all in situation." (p. 174.)

In addition to these distinctions Dr. Stokes (*Dublin Journ.*, vol. x. p. 46,) attaches considerable weight to the slight extent over which attrition sounds are audible. He says they are frequently lost on removing the stethoscope an inch from the situation where they are generated. M. Bouillaud (vol. i. p. 197,) justly remarks that the attrition murmur is "superficial, diffuse, or *peripheric*" in its nature. Another distinction will be found in the accompanying pulse.

Inorganic Murmurs. The section which treats of these has undergone no material alteration. The experiments on dogs continue to form the basis of the arguments, and the murmurs are almost entirely ascribed to anæmia. There are two circumstances which Dr. Hope believes to characterize inorganic murmurs in the heart, which are by no means generally known. The first is that they invariably accompany the systolic movement; the second that they occur only at the aortic orifice. And the explanation of these facts is far from difficult. It is obvious that murmurs cannot be produced in the aortic or pulmonic orifice, during the *diastole*, without the existence of regurgitation, which presupposes *organic* disease; and in regard to the auricular orifices Dr. Hope's cases show that murmurs are very rarely heard, even during the presence of the greatest organic disease. They can only be generated in the arterial orifices, because they are *systolic*, and a systolic mitral or auricular murmur is regurgitant, and must result from organic disease. Laennec stated that inorganic murmur most frequently accompanies the diastole of the heart, but he was, doubtlessly, describing cases of aortic regurgitation; his theory of the diastole, of course, prevented him from looking to the aorta for diastolic murmurs, nor would he necessarily have found very obvious marks of disease if he did, since regurgitation may result from a condition of the valves which does not always present striking indications of its morbid nature, and can only be detected by a person whose previous knowledge leads him to suspect it. Dr. Elliotson was, we believe, the first person who limited inorganic murmurs to the ventricular systole. He says, "the temporary bellows'

sound has always, in my experience, been at the contraction of the ventricles." (*Lum. Lec.* p. 18.) In this, as in many other respects, he was much in advance of the profession in general.

The physical circumstances which produce inorganic murmurs in the heart, the immediate causes on which they depend, the condition of the aortic orifice in an anæmic individual whereby it takes on sonorous vibrations, the real essential difference between it and the ordinary state of the orifice, do not appear to us capable of explanation with our present amount of facts. The only circumstance which is constant in its existence is an excited action of the heart. Dr. Hope would add to this a diminished quantity or attenuated quality of the blood, conditions which certainly occur in the majority of cases, but which are by no means constant or essential. We recently attended the amputation of a leg, in the case of a healthy, robust, agricultural lad of fourteen: during the violent palpitations occasioned by the operation, a loud blowing murmur accompanied the ventricular contraction, which ceased entirely when the operation was over. The accident was a compound fracture, and had certainly induced considerable hemorrhage, but not to such an extent as to render the patient sensibly affected by the loss of blood; nor did he present any of the symptoms of anæmia. Both Andral and M. Meriadec Laennec assign *plethora*, a real bonâ fide increase in the quantity of blood, as an occasional cause of inorganic murmur, and state that the morbid sound may be removed by bloodletting, a sufficient proof that it could not have depended on anæmia. These facts prove, in our opinion, that an excited action of the organ alone is the occasional cause of murmurs, and we do not think sufficient evidence has been collected as to the mediate or immediate nature of the influence exerted by the anæmia when that condition is known to exist. One fact is certain, that before anæmia can occasion a murmur it must bring on excited action.

When an inorganic murmur occurs in an artery, in an anæmic patient, another circumstance which may have some influence in its production, is the flaccid condition of the coats of the vessel. This subject is sufficiently familiar, from the ingenious illustrations it has received in the experiments of Dr. Corrigan. The whole argument is ably treated by Dr. Hope, but we regard it still as incomplete, and beg to point the attention of practitioners to it, as a subject on which the publication of the symptoms and *treatment* of cases would be an exceedingly valuable addition to our knowledge.

Sufficient attention has not been given, in the course of this investigation, to the experiments of John Hunter, by which he endeavoured to determine the relative proportion of muscular and simply elastic contractility possessed by the coats of arteries. He proved, in those experiments, that, when the quantity of blood in a vessel is diminished, the vascular coats do not remain relaxed or "*flabby*," as Dr. Corrigan terms their condition, but that they contract closely around the quantity which remains; that their caliber is sensibly diminished, and that they thus acquire a quality of real *tension*, approximating that of a cord. (*On the Blood*, p. 124, *et seq.*) It is, in our opinion, a question invitingly open to enquiry, whether a vessel in this condition would not be necessarily excited into sonorous vibrations by the jerking action of the heart, on the common principles of acoustics, according to which tension is an almost essential element in the production of sound, and whether this is not precisely the state of things in a case of anæmic palpitations with murmur. The observations of Dr. Hope, Dr. Williams, and the London Committee, on the first sound of the heart, appear to prove that tension is equally necessary to the generation of sound in a hollow body as in a solid string. We offer this as a suggestion to the numerous enquirers who are so actively engaged in the investigation of this subject.

Venous Murmurs. Under this head the author has added a section containing an elaborate description with many interesting original observations, if the "continuous murmur" of Laennec, or the "*bruit de diable*" of Bouillaud, which was first shown by Dr. Ogier Ward, of Shrewsbury, to be seated in the veins. This murmur almost, if not quite invariably results from anæmia, of which it is a valuable sign. We are inclined to place much confidence in the constancy of its presence as an attendant on this obscure and deceptive disease. In those cases of anæmia, and they are very numerous, which so closely similate hyperæmia as to create perplexing doubts of their real nature, we anticipate that the venous murmur will be found an efficient aid in diagnosis. Dr. Hope states that he has never found the murmurs to exist, in any marked degree, in any instance in which anæmia was absent. Further, he has invariably found that they gradually disappeared in proportion as the anæmic state was removed by iron, aloetic aperients, animal food, and fresh air, (p. 122-4.) These results are in exact accordance with those of M. Bouillaud, and we have much pleasure in adducing the further testimony of our own observation to their truth.

We transcribe some of the more interesting of Dr. Hope's observations, which our readers will find no difficulty in repeating, on the greater number of their anæmic or chlorotic patients.

"The venous murmur is on a much lower key than the arterial murmur, for, while the latter is often as high as the note produced by whispering the letter *r*, and seldom lower than *au*, the venous murmur is usually as low as *who*. This sound, indeed, offers the most complete and ready imitation of the phenomenon with which I am acquainted. The hollow sound of a large incessant forge bellows also imitates it very closely. When the arterial throb is considerable, the murmur experiences augmentations corresponding with each arterial diastole or pulse." (p. 110.)

"When the vein under examination is very superficial, merely subcutaneous, as the external jugular, very light pressure with the stethoscope will increase the murmur by partially contracting the caliber of the vessel; but if the vein be obliterated by laying the point of the finger lightly upon it above the stethoscope, or by depressing the upper edge of the stethoscope, the murmur instantly ceases." (p. 111.)

"Strong pressure with the stethoscope, sufficient to obliterate a subcutaneous vein, as the external jugular, if applied on the internal jugular, instead of suspending the murmur, swells it *gradually* to a surprising degree of intensity and diffuseness, like blowing the word *who* with great force, mixed up with which sound I have frequently heard humming, cooing, and whistling notes, appearing to proceed from several veins at once, which I shall hereafter show to be the case." (p. 112.)

"If the point of a finger be nicely dropped on the internal jugular vein in any part of its course, so as to obliterate the vessel, yet without obliterating the carotid, the loud murmur instantly ceases, and nothing is heard but a dull, obscure rumbling seated in smaller veins. It is occasionally mixed up with puny, humming, and whistling notes. If the finger be now raised again from the internal jugular, the torrent rushes down and restores the original loud murmur almost as promptly as when the finger is raised from the hole of a wind instrument. By alternately raising and depressing the finger, the most sceptical may soon convince himself that the seat of the murmur is really in the vein. One or two precautions are required. If the neck be displaced from the perpendicular, the sterno-mastoid muscle is apt to be put so much on the stretch as to obliterate the internal jugular and suspend the murmur. Again, if the stethoscope, placed behind the sterno-mastoid, press that muscle too much forward, it will obliterate the internal jugular. Again, if the skin be stretched across the neck, under the stethoscope, the tension will increase the murmur in most; but in a few, whose internal jugular is very superficial, it will obliterate the vessel and suspend the murmur." (pp. 112-13.)

"The loud murmur of the internal jugular becomes louder during inspiration, especially about its end, and weaker during expiration. I have noticed that when an anæmic patient becomes faint from long standing under examination, the murmur, previously loud and constant, becomes extinct except during the inspirations. This evidently proceeds from a deficient afflux of blood to the head, whence there is not a sonorous current down the veins, except when it is favoured by the suction of inspiration. For the latter reason, the murmur exists during inspiration only, in those who barely exhibit the phenomenon at all, for instance, the convalescent from anæmia." (p. 114.)

"In order to produce a murmur of the internal jugular in perfection, it is necessary to avert the face, while the neck is kept perpendicular and the chin well raised. When the head is restored to its natural position or is depressed, the murmur ceases or greatly diminishes." (p. 115.)

"As the respiratory murmur simulates the venous murmur, learners should request the patient to hold his breath." (p. 116.)

The venous murmur occasionally assumes a musical character, which Dr. Hope proposes to denominate the "venous hum;" it frequently resembles very closely a smart wind blowing through a keyhole. Its indications are of no specific nature.

Of Musical Murmurs we shall add nothing to what we have already said. They are merely modifications of common murmurs, and cannot be referred to any distinct or peculiar morbid action.

Abdominal Murmurs. A valuable chapter is introduced on auscultation applied to pregnancy, divided into two parts. In the first the rules for exploring the pulsations of the fœtal heart are concisely and practically explained, in a manner which will be found useful to the obstetric student. In the second, the author enters into an examination of the doctrine of utero-placental murmur, in the course of which he develops some new views on this imperfectly understood phenomenon, and endeavours to determine its real nature and value. He submits for further investigation the following propositions:

"1. That the murmur is arterial when it is a whiff. 2. That it is venous when it is continuous without augmentations synchronous with the pulse. 3. That it is arterial and venous conjoined when it is continuous with augmentations. 4. That its seat is sometimes in the vessels of the abdominal parietes, as the epigastric, circumflex ilii, internal mammary, and their branches and concomitant veins; sometimes in the great arteries and veins within the cavity of the abdomen, as the common and external iliacs, the renal, the three branches of the cæliac, the colica dextra, media, sinistra, and ileo-colica, and the portal veins; sometimes, possibly, in the uterine walls, and sometimes, possibly, in the vessels of various tumours. 5. That the murmur is generally created by pressure, whether that of the uterine or other tumour or of the stethoscope; and that it does not exist independent of pressure, except, possibly, in anæmic cases. 6. That the stretched condition of the arteries, and especially the veins of the abdomen, is favorable to the operation of pressure in producing the murmur." (p. 133.)

He remarks that these propositions cannot be adequately comprehended except by one who is thoroughly imbued, both theoretically and practically, with the doctrines of venous murmur. He then proceeds to demonstrate the analogy between abdominal murmurs and those heard in the neck of an anæmic patient, by quoting the descriptions of the former given by Laennec, Kennedy, and Montgomery. He shows that both are sometimes a mere arterial whiff; sometimes continuous with augmentations; sometimes continuous with little or no augmentations; sometimes ceasing with no assignable reason; and most marked in anæmic subjects. In illustration of these principles, he cites nine cases, which prove that the whole of these murmurs may exist independent of pregnancy. Finally, he shows the inconsistencies and imperfections in the arguments which have been employed to localize these sounds in the placental arteries, and concludes with the three following practical rules. 1. That a near-sounding high-toned murmur may result from any tumour, may exist wholly independent of tumour, and occurs almost exclusively in the thin blooded or anæmic, with a quick pulse. 2. That a distant low-toned murmur, heard on a tumour in the hypogastric region, affords presumptions that the tumour compresses the hypogastric artery. 3. That these murmurs afford presumptions of pregnancy only when they coincide with other signs. We request the reader to compare these observations with the third article in our present Number, p. 365.

Purring Tremor. A concise synopsis is given of the circumstances under which this phenomenon is developed in the heart and arteries. Dr. Hope states that he has observed regurgitation through the mitral valve to be beyond comparison the most frequent cause of tremor in the heart. He has never known it to exist in the heart independently of organic disease. In the arteries it may be produced by disease of their internal coats. Tremor, is, also, occasionally propagated as far as the carotid and subclavian arteries, by considerable contraction of the aortic orifice, but rarely, if ever, propagated so far as the radials. Aortic regurgitation may propagate a tremor as far as the radials, or even to still more remote arteries. Anæmia may, during nervous excitement, produce arterial thrill, without organic disease. In conclusion, as tremor has the same origin as bellows', musical, and other murmurs, it is always accompanied by them; but, as it requires a greater degree of vibration for its sensible development, they may exist without being accompanied by it.

Endo-pericarditis. The general history of this complex disease has undergone valuable revision in the present edition, and has received many useful additions in the form of notes. The morbid appearances of redness, effusion, and adhesion are elaborately described, and the relation of their varieties with the modifications observed in the general symptoms is pointed out. The general signs of the disease, their obscurity, their complexities, their diversities, their inconstancy, the causes of these apparent anomalies, and their relation to diagnosis, prognosis, and treatment are fully discussed. Dr. Hope concurs with Dr. Stokes and Dr. Latham in assigning the greatest importance in the production of these differences to the quantity of effusion in different cases. He combats, and with complete success, the opinion of M. Bouillaud, that these modifications are always attributable to pleuritic or peripneumonic complications. In speaking of the worst train of symptoms induced by the compression of fluid occasioning considerable obstruction to the circulation through the heart, Dr. Hope observes that the same class of symptoms is produced under whatever circumstances the circulation through the heart is greatly impeded; and adds that he has seen them result from poisoning by arsenic and by intense gastro-enteritis; that they result also from poisoning by the mineral acids, tobacco, &c., all of which agents have a paralyzing effect on the heart. He has likewise seen them occasioned by polypi forming in the heart before death and by extreme softening of the organ.

Dr. Hope describes the physical signs of endo-pericarditis under the heads *percussion, impulse,* and *sounds.* In respect of the two former, nothing new is added. He arranges the sounds into two classes: the sounds of the first class, being *direct* signs of pericarditis, arise from the condition of the pericardium itself; the second class are valvular murmurs resulting from endocarditis, which constitute *indirect* signs of pericarditis. The account of the attrition sounds coincides with that of Stokes and Bouillaud; but, in addition to the generally described phenomena, which it is unnecessary to notice here, the author notices a "continuous rumble," which he has occasionally heard, and which he attributes to the agitation of a small quantity of fluid in the cavity of the pericardium.

"In one case, in which the fluid originally caused dulness as high as the second rib, the rumble came on with tremor, when the quantity of fluid became moderate; it passed into a double attrition sound with tremor, when the fluid underwent further absorption, and both phenomena ceased, when complete cessation of dulness and other signs indicated complete adhesion of the pericardium, which I ascertained to have taken place by post-mortem examination a year and a half afterwards." (p. 167.)

Murmurs of the second class, or those indicative of endocarditis, are identical with the valvular murmurs, of which we have given so ample an account. The three signs which, according to Dr. Hope's experience, are the least number that suffice to indicate inflammation of the heart, are increased action of the heart, fever, and a murmur which did not previously exist.

Connexion of Diseases of the Heart with Apoplexy, Palsy, &c. Dr. Hope's experience goes strongly to confirm the old Italian doctrine on this point. In illustration of it he has given an analysis of thirty-nine cases, treated in the Marylebone Infirmary, to which we beg to direct the attention of our readers.

Partial Dilatation of the Heart. The chapter on this subject is principally devoted to an abstract of Mr. Thurnam's valuable paper from the Medico-Chirurgical Transactions for 1838. Some interesting and instructive facts are adduced relative to this obscure affection, but they do not throw much light on either its diagnosis or its treatment, as distinguished from other organic diseases of the heart. The obscurity of this disease should render it an especial object of attention to the profession; it offers to the enquiring practitioner an inviting field for original research. Dr. Hope suggests that every novelty or anomaly, either in character or situation, of a murmur, and of the accompanying pulse and impulse, should be accurately noted and compared with post-mortem appearances, in order more clearly to determine the phenomena, if any there be, peculiar to real aneurism of the heart.

Diagnosis of Softening. Much useful matter is added to this chapter, and we are induced to hope that softening will henceforth take its place among the diseases which a stethoscopic exploration can distinguish with tolerable facility and general accuracy. Dr. Hope's contributions to our means of detecting this affection will be highly appreciated, when it is remembered that the latest writer on the subject, M. Bouillaud, has scarcely given any diagnosis at all. In addition to the general symptoms of organic disease, softening is indicated, according to our author, by a very weak and sometimes imperceptible impulse, as was stated by Laennec, by both sounds being weaker than natural, and the first sound becoming short and flapping like the second, and by irregular, unequal, feeble, and intermittent pulse, without valvular murmur. Some cases, illustrative of these points, are inserted at the end of the chapter.

Signs of Adipose Disease of the Heart. This disease is also illustrated with cases which lead to the inference that fat impedes the action of the heart and obstructs the circulation; and that its signs, so far as Dr. Hope can judge, are, 1, diminution of the sounds, especially the first, without the flap of softening; 2, irregular pulse without valvular disease; 3, "oppression" or even pain in the precordial region, with general signs of a retarded circulation, producing cerebral, hepatic, and other congestions.

Thoracic Aneurism. In an appendix to the chapter on aneurism of the aorta are related two cases. The first is a case of aneurism of the aorta bursting into the right ventricle, and leads to the presumption that the signs of this disease are: *a*, a remarkably loud, harsh, superficial sawing murmur, with both the systole and diastole, together with a continuous *incessant* rumble; both most audible above the level of the fourth rib, on or near the sternum, and thence, along the tract of the pulmonary artery, up the interspace between the second and third rib; *b*, a purring tremor in the same situations; *c*, weakness or extinction of the second sound, in consequence of the reaction of the aortic blood on the valves being enfeebled by the regurgitation; *d*, pulse preeminently jerking; *e*, great and rapid dropsy; *f*, livid complexion; *g*, the evidence is stronger if the symptoms followed a lift or effort producing faintness and paleness. The second case is one of rupture of a dilated aorta into the pulmonary artery, of which the following were the signs observed: *a*, a very loud, superficial, sawing murmur, prolonged continuously over the first and second sounds (and probably weaker during the interval of repose), loudest along the tract of the pulmonary artery; *b*, a purring tremor in the pulmonary artery, in the interspace between the second and third ribs; *c*, the second sound weakened at the clavicles; *d*, the jerking pulse; *e*, great, rapid, and universal dropsy; *f*, a livid, venous tint of surface; *g*, the symptoms following an effort.

Abdominal Aneurism. On the physical diagnosis of this disease much new and valuable matter is introduced. After describing the physical signs of the aneurism, the tumour, pulsation, dulness on percussion, and bellows' murmur, Dr. Hope proceeds to point out the fallacies which may arise from the existence of various abdominal tumours, and the means by which they may be detected and obviated. An abdominal tumour, he states, may generally be distinguished from aneurism of the abdominal aorta by the following characters: 1. The impulse is more feeble, especially if the tumour be large or superficial. 2. The impulse is rendered still more feeble by applying the stethoscope laterally. 3. The tumour is often moveable with the viscus in which it is seated. 4. The tumour and impulse may often be removed by a brisk purgative. 5. Tumours are generally less compressible than aneurisms. 6. In enlargement of the liver, dulness extends from the right hypochondrium, *without any interval*, to the seat of the pulsation. 7. The murmur of a tumour is generally less than that of an aneurism. 8. Collateral and important evidence may be derived from the general history of the case. The author subjoins a case in which a fatal aneurism produced very few of its characteristic phenomena, and was indicated solely by epigastric pulsation. Another source of fallacy consists in the pressure of the stethoscope, which may generate a murmur in a superficial artery situated over the tumour. Such a murmur will be distinguished by its *nearness*, its hissing tone, its being restricted to one spot, and by its ceasing when the vessel is obliterated by firm pressure.

Nervous Affections. The chapters on angina pectoris and syncope remain as in the original edition. That on nervous palpitation is very considerably enlarged and modified. Inorganic palpitation is divided into the following four varieties, according to its cause: 1. Palpitation excited by various derangements of the nervous system only. 2. Palpitation from anæmia. 3. Palpitation from too stimulant diet. 4. Palpitation from plethora. As the first, third, and fourth varieties do not occasion murmurs, they are distinguishable from organic disease without much difficulty by a practised auscultator. Nor will much difficulty be encountered in establishing the diagnosis of anæmic palpitations, by an auscultator who is conversant with the rules laid down in this work for differential valvular diagnosis.

Displacements. The following cases of murmurs from displacement are interesting:

"I at present attend a young lady, Miss M., in whom the heart was forced entirely over to the right of the sternum by pleuritic effusion in the left pleura. The aorta was felt to pulsate between the second and third right ribs, an inch from the sternum, and here a murmur was heard with the first sound, which has ceased since the heart has been restored to its natural situation by the absorption of the fluid. Is it therefore possible that a twist given to the aorta, or pressure of the vessel against the ribs, may be the cause of a murmur under such circumstances?

"I have at present two cases of still greater displacement of the heart to the right, in consequence of universal consolidation and contraction of the right lung and hypertrophy of the left. The ascending aorta beats between the second and third right ribs, two and a half inches from the sternum in one case, and one and a half to two in the other. There is a murmur with the second sound, from aortic regurgitation, in the former case. It remains to be seen whether regurgitation proceeds from a twist in the aorta, disabling the valves, or from disease of the valves themselves. The pulsation of the aorta so far on the right, might be, and actually was, mistaken by non-auscultators for an aneurism.

"When the heart is displaced to the right just so far as to be impacted between the sternum and the spine, I have found its impulse to be considerably increased, so as to convey the idea of hypertrophy. This occurred in the case of Miss M. above described, and, until I pointed out the circumstance, the disease was mistaken for hypertrophy, the pleuritic effusion being overlooked." (pp. 536-7.)

Pulses of Disease of the Heart. We have noticed all that is new in the text, on this subject, in the remarks we have made on valvular diagnosis, softening, and adipose disease. A table is, however, appended to the work, containing a synopsis of the characteristic pulse of every disease of the organ, which, although still very imperfect, will be found useful for reference: we therefore transcribe it.

*Table of Pulses of Disease of the Heart.*

"Simple Hypertrophy of Left Ventricle. Pulse *strong* and *tensely prolonged*, because the ventricle contracts powerfully but slowly.

"Hypertrophy with Dilatation. *Strong*, *tensely prolonged*, and *large*, because the ventricle contracts powerfully, slowly, and expels an increased quantity of blood.

"n.b. If the above pulses be moderately accelerated, they become '*hard*.' They may be rendered temporarily or permanently *small* and *weak* by any debilitating causes impairing the contractile power of the heart. Also by extreme palpitation and dyspnœa causing engorgement of the organ.

"Hypertrophy with Contraction. *Tense but small*, and if the contraction be considerable, it becomes weak as well as small, from the insufficient quantity of blood propelled into the arteries.

"Dilatation with Hypertrophy, i. e. the dilatation being predominant. *Large* and rather *prolonged* but *soft*, from the larger capacity, but weakness of the ventricle.

"n.b. This pulse if accelerated becomes 'bounding.'

"Dilatation with Attenuation. *Large* and *weak*, becoming *small* in the last stage, when the ventricle is too weak to expel its contents.

"Softening. *Small*, *weak*, and more or less *irregular*, *unequal*, and *intermittent*, sometimes extremely so, in the late stages, from the debility of the ventricle.

"Free Regurgitation through the Aortic Valves. Eminently *jerking;* from the arteries being unfilled.

Contraction of the Aortic Valves. *Strength* little impaired unless the contraction be very considerable. The *regularity is seldom affected*, except by extreme contraction.

"Great Contraction of or free Regurgitation through the Mitral Valve. *Small*, *weak*, *irregular*, *intermittent*, and *unequal*, because contraction occasions an insufficient and irregular supply of blood to the ventricle; and because regurgitation weakens the pulse, in consequence of the resistance of the mitral valve being removed, and disturbs its regularity in consequence of rendering the supply of blood less uniform.

"A large Polypus formed before death. Suddenly causes a small, weak, irregular, and intermittent pulse, because the polypus chokes up the ventricle.

"Endocarditis with Polypus. Ditto.

"Pericarditis with much serous Effusion compressing the Heart. Ditto." (pp. 623-4.)

The sixth part of the work contains a valuable collection of cases. A few of the less complete cases in the former edition have been removed, and others of an interesting nature, illustrating particular points for the most part new, have been added.

The plates will be found a valuable improvement in the present edition. They illustrate most of the diseases of the heart which are difficult of comprehension. The frontispiece, which is a sketch of the heart in situ, exhibiting its relation to the ribs and surrounding parts, will greatly facilitate the student's labour in the practical prosecution of his studies. The plates reflect great credit on Dr. Hope as an artist, and they are neatly engraved.

In concluding our remarks on this work, we wish to observe that we hope none of our readers will imagine, because our attention has been chiefly directed to the portion of it relating to physical diagnosis, that we are either disregardful of the advantage and necessity of combining an intimate knowledge of the general symptoms of diseases with that of auscultation, or that the work contains an imperfect account of the anatomy, pathology, causes, general symptoms, treatment, &c. of the different diseases. On the contrary, it is our opinion that a principal advantage conferred on practical medicine by the discovery of auscultation consists in the closer attention to the general history of diseases, and the more correct appreciation of their respective general symptoms, which its study has necessarily induced. Without having thoroughly studied this part of Laennec's discovery, an auscultator is little superior, in the facility and precision of his diagnosis, to an experienced non-auscultator. Our reason for having so slightly referred to this subject is the obvious one, that we are reviewing, not the whole work, but the additions to the former edition of it. Ere long we may probably take up the whole subject of diseases of the heart; when we shall have occasion to compare Dr. Hope's treatise with some others recently published on the continent. We cannot, however, close the present notice without observing of the work, taken as a whole, that it is, in our opinion, by far the best publication on diseases of the heart and great vessels that exists in this or in any other language; an estimate grounded as much on the history which it contains of the general nature, course, and treatment, as of the physical diagnosis of the several diseases. No author evinces a more philosophical discrimination in his valuation of the relative importance of general and physical signs in establishing a diagnosis than Dr. Hope; and no writer has more fully and judiciously availed himself of the assistance of the former class of signs. On this point, indeed, we cannot better state our own opinion than by quoting that of the author, as expressed in the concluding paragraph of his introduction.

"With respect to the comparative value of the general and physical signs of disease of the heart, it may be said that Laennec rather undervalued the former and overrated the latter. This was owing principally to the general signs being less perfectly understood when he studied than they have subsequently become *in consequence of being investigated with the aid of auscultation.* The ardour of his early disciples, who imagined that the physical rendered the general signs superfluous, brought auscultation into some disrepute by the inaccuracy of their diagnosis. But since the stethoscope has taken its proper place *as an auxiliary only,* and the diagnosis has been founded on the two classes of signs conjointly, auscultation has ranked as a discovery which will immortalize its author and form an epoch in the history of medicine."

---

